# Left atrial sphericity in relation to atrial strain and strain rate in atrial fibrillation patients

**DOI:** 10.1007/s10554-023-02866-2

**Published:** 2023-05-31

**Authors:** Luuk H.G.A. Hopman, Pranav Bhagirath, Mark J. Mulder, Ahmet Demirkiran, Sulayman El Mathari, Anja M. van der Laan, Albert C. van Rossum, Michiel J.B. Kemme, Cornelis P. Allaart, Marco J.W. Götte

**Affiliations:** 1 Department of Cardiology, Amsterdam UMC, Vrije Universiteit Amsterdam, Amsterdam Cardiovascular Sciences, Amsterdam, The Netherlands; 2 Department of Cardiothoracic Surgery, Amsterdam UMC, Amsterdam, The Netherlands; 3 Dept. of Cardiology, Amsterdam UMC, De Boelelaan 1118, 1081 HV Amsterdam, The Netherlands

**Keywords:** Cardiac MRI, Atrial remodeling, Atrial fibrillation, Atrial strain, Atrial sphericity

## Abstract

**Purpose:**

Left atrial (LA) sphericity is a novel, geometry-based parameter that has been used to visualize and quantify LA geometrical remodeling in patients with atrial fibrillation (AF). This study examined the association between LA sphericity, and LA longitudinal strain and strain rate measured by feature-tracking in AF patients.

**Methods:**

128 AF patients who underwent cardiovascular magnetic resonance (CMR) imaging in sinus rhythm prior to their pulmonary vein isolation (PVI) procedure were retrospectively analyzed. LA sphericity was calculated by segmenting the LA (excluding the pulmonary veins and the LA appendage) on a 3D contrast enhanced MR angiogram and comparing the resulting shape with a perfect sphere. LA global reservoir strain, conduit strain, contractile strain and corresponding strain rates were derived from cine images using feature-tracking. For statistical analysis, Pearson correlations, multivariable logistic regression analysis, and Student *t*-tests were used.

**Results:**

Patients with a spherical LA (dichotomized by the median value) had a lower reservoir strain and conduit strain compared to patients with a non-spherical LA (-15.4 ± 4.2% vs. -17.1 ± 3.5%, *P* = 0.02 and − 8.2 ± 3.0% vs. -9.5 ± 2.6%, *P* = 0.01, respectively). LA strain rate during early ventricular diastole was also different between both groups (-0.7 ± 0.3s^− 1^ vs. -0.9 ± 0.3s^− 1^, *P* = 0.001). In contrast, no difference was found for LA contractile strain (-7.2 ± 2.6% vs. -7.6 ± 2.2%, *P* = 0.30).

**Conclusions:**

LA passive strain is significantly impaired in AF patients with a spherical LA, though this relation was not independent from LA volume.

**Supplementary Information:**

The online version contains supplementary material available at 10.1007/s10554-023-02866-2.

## Key points

- This study found that LA passive function, measured using strain assessment, is significantly impaired in AF patients with a spherical LA as compared to patients with a non-spherical LA.
- The relation between LA sphericity and LA strain was not independent from LA volume.
- In patients with a spherical LA, an increase in LA pressure is related to a deterioration in LA function, while in patients with a normal non-sphere shaped LA, LA function remains largely preserved.

**Supplementary Information:**

The online version contains supplementary material available at 10.1007/s10554-023-02866-2.

## Background

Recent studies have demonstrated the importance of left atrial (LA) geometry on persistence of atrial fibrillation (AF), as well as on recurrence risk after AF ablation [1–5]. One of the important geometry-based markers is LA sphericity, a measure that quantifies the difference between the shape of the LA and a perfect sphere [6]. Spherical remodeling would be a geometrical adaptation to cope with an atrial pressure overload [6]. This LA morphological transformation (i.e. spherical remodeling) might as well impact LA function, although this relationship has not yet been assessed in detail. Moreover, research on the contribution of LA pressure to geometrical and functional LA remodeling is currently limited.

Cardiovascular magnetic resonance (CMR) myocardial feature tracking (FT) has proven to be a feasible and reproducible technique for the evaluation of LA deformation. FT strain can be used to assess all phases of LA function including the reservoir, conduit, and the contractile phase [7, 8]. Strain and strain rate provide information about the LA expansibility, stiffness, and contractile function [9, 10], which all may be related to LA spherical remodeling.

This study investigated the relationship between LA sphericity, intra-atrial pressure, and LA phasic function assessed using strain and strain rate.

## Methods

### Study design

This retrospective single-center study was conducted in accordance with the Declaration of Helsinki. The local medical ethics committee (Amsterdam UMC, location VU University Medical Center, Amsterdam, The Netherlands) approved the study protocol and all patients provided written informed consent. The study population comprises a cohort of consecutive patients that underwent CMR prior to first ablation for AF.

### Study population

Between July 2018 and June 2021, 133 consecutive AF patients were enrolled [11]. All patient were scheduled for a first-time pulmonary vein isolation (PVI) radiofrequency ablation. Prior to this PVI procedure, patients underwent CMR imaging for the assessment of cardiac function and pulmonary vein (PV) anatomy as part of clinical routine.

Exclusion criteria were general CMR contraindications, contraindications for a gadolinium-based contrast agent, a cardiac implantable electronic device, mechanical heart valves, and absence of sinus rhythm during CMR. Therefore, all patients included in the study were in sinus rhythm during the MRI scan, irrespective of whether they had been diagnosed by the referring physician with paroxysmal or persistent AF.

In a subset of patients, LA pressure measurements were performed during the ablation procedure.

### CMR Protocol

A detailed protocol with the specific CMR parameters used has previously been described [12]. Briefly, images were acquired using a 1.5 Tesla magnetic resonance imaging system (Siemens AVANTO or SOLA, Erlangen, Germany) and a 32-channel array coil. The CMR protocol consisted of steady state free precession cine imaging in long axis orientations (two-chamber and four-chamber view) and an electrocardiogram gated free-breathing navigator-based 3D contrast enhanced magnetic resonance angiogram (CE-MRA).

### CMR data analysis

#### LA volume and function

Analysis of cine images was performed using Circle CVI^42^ (Version 5.11, Circle Cardiovascular Imaging, Inc, Calgary, Canada). Using the biplanar method, volumetric data of the LA and LV were derived from two-chamber and four-chamber cine images. LA volume (LAV) was divided in minimal (LAV_min_) and maximal (LAV_max_). From these volumes, the total LA emptying fraction (LAEF) was derived. LAV index maximum (LAVi_max_) was calculated by dividing LAV_max_ by body surface area.

#### LA strain assessment

LA strain analysis was performed using Circle CVI^42^ Feature Tracking software (Version 5.11, Circle Cardiovascular Imaging, Inc, Calgary, Canada). Endocardial and epicardial borders were manually traced in the end-systolic phase of the long-axis two-chamber and four-chamber cine images, which sets the ventricular end-systole as a zero-point for LA strain analysis. An automated tracking algorithm was used and manual adjustments were applied as needed to attain optimal wall tracking.

**Fig. 1:**
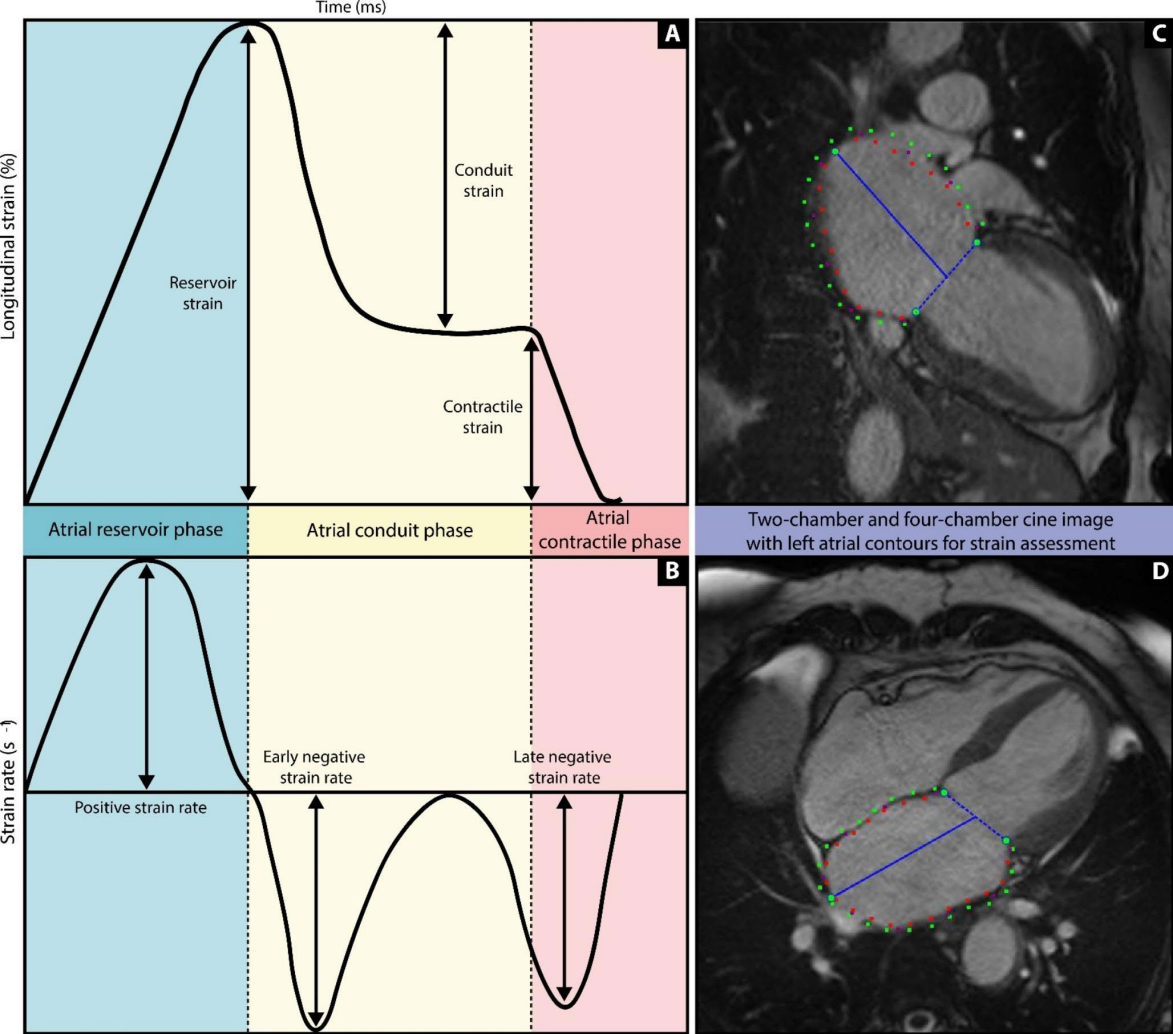
Illustration explaining CMR feature tracking derived phasic strain and strain rate curves **(A)** Illustration of a LA feature tracking longitudinal strain graph demonstrating the different phases of LA function. **(B)** Illustration of a LA feature tracking longitudinal strain rate graph. Feature tracking strain requires a left atrial endocardial and epicardial contour in the end systolic phase in the 2-chamber **(C)** and 4-chamber **(D)** cine images. An advanced post-processing technique tracks the LA wall over time

Longitudinal strain measurements were subdivided into LA reservoir strain, conduit strain and contractile strain. Furthermore, LA positive strain rate (SRs), LA early negative strain rate (SRe), and LA late negative strain rate (SRa) were derived from strain rate curves. An illustration of LA strain analysis is shown in Fig. 1.

#### LA sphericity assessment

**Fig. 2:**
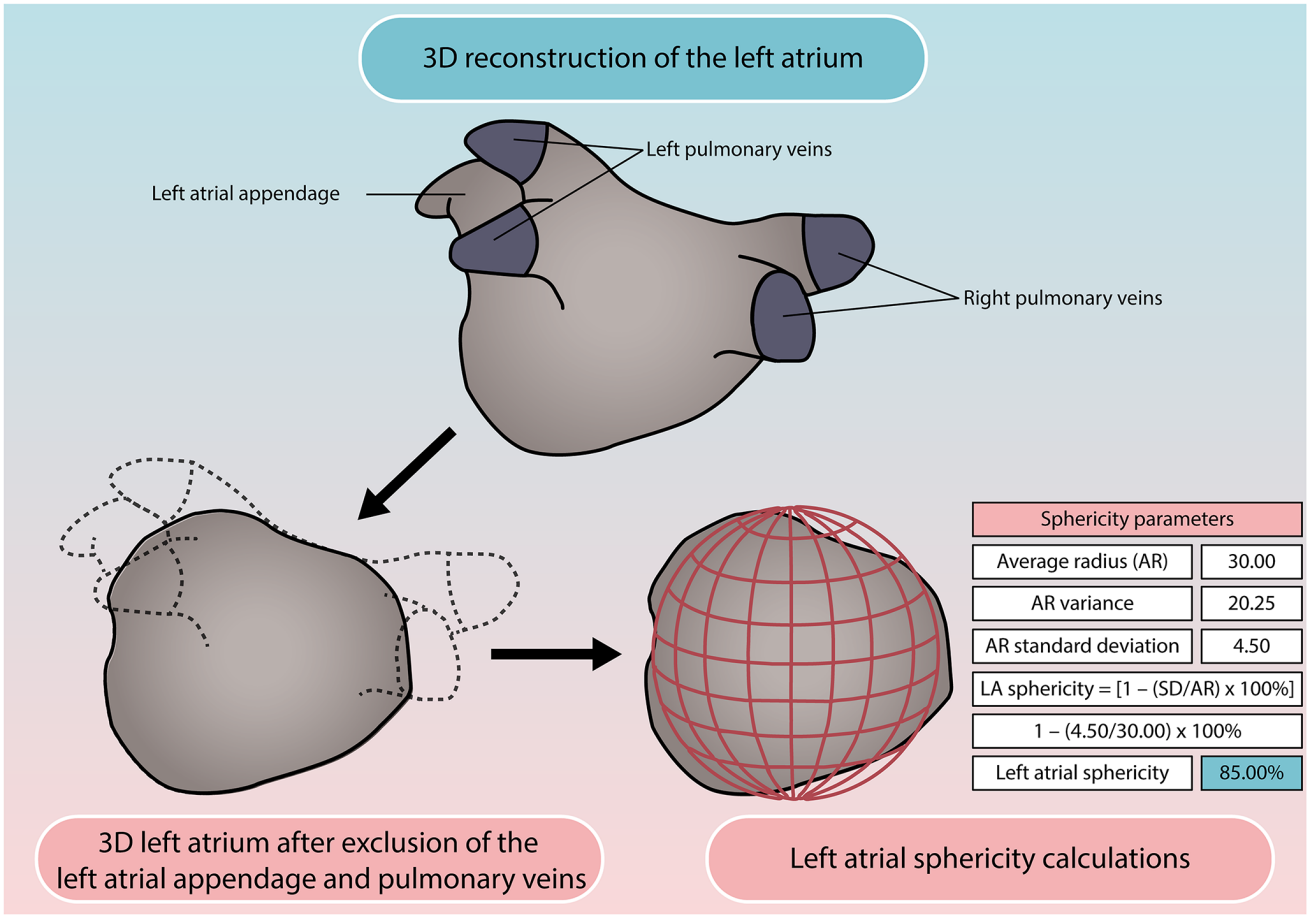
Illustration explaining the LA sphericity calculation A 3D reconstruction of the LA can be made using dedicated segmentation software. Thereafter, the pulmonary veins and LA appendage are excluded at their ostia, defined as the site of reflection of these structures with the LA wall. CEMRG software was used to automatically calculate LA sphericity. This software evaluates the variation between the LA and a sphere that best fits the LA shape. In short, the center of mass of the LA was determined. Hereafter, the average radius between all points of the LA wall and the center of mass was calculated. The average radius (AR) represents the radius of the sphere that best fits the LA. The AR standard deviation (SD) and AR of the distances between all points of the LA wall and the center of mass are used to calculate the LA sphericity with the formula [1 – (SD/AR) x 100%]

Calculation of LA sphericity was performed using open source software (CE-MRG (Cardiac Electro-Mechanics Research Group), King’s College London, United Kingdom) [13]. Using a thresholding tool, the LA blood pool and PV extensions were segmented semi-automatically in the 3D CE-MRA images on axial slices. The interpolated contours were adjusted manually if deemed necessary in each axial plane. A 3D reconstruction of the LA was generated and thereafter, both the PVs and LA appendage were excluded at their ostia, defined as the site of deflection from the LA wall. The mitral valve annulus was used as landmark to separate the LA from the LV. A 3D volume was derived from the LA cavity segmentation. The 3D LA segmentation was also used to calculate LA sphericity using the algorithms published by Bisbal and colleagues [6]. In this regard, a sphericity of 100% represents a perfect sphere, whereas non-spherical shapes will have lower values (Fig. 2).

### LA pressure measurement

LA pressure was measured via a trans-septal sheath (8.5 F, SL0, Abbott, St. Paul, MN, USA) in a subset (n = 76) of patients during the PVI procedure while patients were in sinus rhythm (post-procedural pressure). None of the patients were under general anesthesia. The trans-septal sheath was connected to a pressure transducer and recorder (Xper IM, Philips Healthcare, Best, The Netherlands). The maximum LA pressure (LAPmax) was defined as the maximum height of the v wave, and the minimum LA pressure (LAPmin) was defined as the minimal value of the x-wave during measurement. Mean LA pressure (LAPmean) was defined as (LAPmin + 1/3(LAPmax − LAPmin)).

### Statistical analysis

Results are presented as mean ± standard deviation (SD) for normally distributed data and median including interquartile range (IQR) for data with a non-normal distribution. Normality of continuous data was assessed by inspection of histograms and Q-Q plots. To test for differences between two groups the Student *t*-test or Mann-Whitney U test was used, as appropriate. Pearson’s correlation was used to quantify associations between continuous variables. To identify independent predictors of LA strain, multivariable linear regression analysis was performed. Intra- and inter-observer variability of LA sphericity measurements were assessed by intraclass correlation coefficients (ICC) for absolute agreement based on two-way random model. Data were considered significant if *P*-value < 0.05. Statistical analysis was performed using SPSS Statistics v26 (IBM Corporation, Armonk, NY, USA).

## Results

### Patient characteristics

Good quality cine images were available in 92% of AF patients (122/133) and good assessable 3D CE-MRA images for quantification of LA sphericity were available in 128/133 patients (96%). The baseline characteristics of the study population are presented in Table 1. In the study cohort, mean age was 60 ± 10 years and 62% were male. The median duration between diagnosis of AF and CMR scan was 25 months (13–65 months). The median time between CMR scan and pressure measurements during PVI was 28 days (15–83 days).

**Table 1:** Baseline characteristics of the study population

	*n* = 133
*Demographics*	
Age, years	60 ± 10
Male gender	83 (62%)
Height (cm)	179 ± 10
Weight (kg)	83 ± 14
BMI (kg/m^2^)	25.7 ± 3.5
BSA (Mosteller)*	2.0 ± 0.2
CHA_2_DS_2_-VASc score ≥ 2	47 (35%)
Hypertension	45 (34%)
Diabetes mellitus	5 (4%)
History of stroke/TIA	4 (3%)
Congestive heart failure	13 (10%)
Presence of mitral valve insufficiency	45 (37%)
Grade I	40 (32%)
Grade II	3 (2%)
Grade III	2 (2%)
AF duration (months)	32 [12–78]
*Medications*	
ACE inhibitor or ARB	37 (28%)
Beta-blocker	37 (28%)
Amiodarone	14 (11%)

Values are expressed as number (percentage), mean ± SD or median [25th-75th percentile]. ACE, angiotensin-converting-enzyme; ARB, Angiotensin-receptor-blocker; AF, atrial fibrillation; BMI, body mass index; BSA, body surface area; CHA_2_DS_2_VASc, history of congestive heart failure, hypertension, diabetes mellitus, stroke/transient ischemic attack/prior thromboembolism, vascular disease, age and sex; CMR, cardiovascular magnetic resonance; TIA, transient ischemic attack. *Calculated by the Mosteller method ((height (cm) x weight (kg)/3600)^½^)

### LA sphericity

Mean LA sphericity was 79.22 ± 3.13% and similar between patients with and without presence of mitral insufficiency (MI) (grade ≥ 1) on echocardiography (79.15 ± 3.10% vs. 79.27 ± 3.22%, *P* = 0.84, respectively). LA sphericity was significantly higher in patients with hypertension compared to patients without (80.00 ± 3.11% vs. 78.81 ± 3.07%, *P* = 0.04, respectively).

### LA volume and strain

LA volumetric and functional parameters are listed in Table 2. 3D LAV was 104.44 ± 30.43mL and LAEF 52.39 ± 13.25%. 3D LAV was inversely correlated with both LAEF and LA strain (r=-0.51 and r=-0.55, *P* < 0.001 for LAEF and LA reservoir strain, respectively), and correlated with LA sphericity (r = 0.39,*P* < 0.001). Mean LA reservoir strain, conduit strain and contractile strain were − 16.22 ± 3.95%, -8.86 ± 2.85% and − 7.36 ± 2.43%, respectively.

**Table 2:** Differences in LA and LV parameters in patients with a non-spherical LA and spherical LA

	Non-spherical LA (≤ 79.13%) *n* = 61	Spherical LA (> 79.14%) *n* = 61	*P*-value
*LA volume*			
3D LA volume (ml)	96.09 ± 23.42	112.92 ± 34.34	**< 0.01**
LA volume index - min (ml/m^2^)	19.19 ± 8.28	28.45 ± 15.40	**< 0.001**
LA volume index - max (ml/m^2^)	41.89 ± 11.43	54.38 ± 14.90	**< 0.001**
LA stroke volume (ml)	45.68 ± 13.36	53.27 ± 18.19	**0.01**
LA emptying fraction (%)	55.11 ± 11.03	49.82 ± 14.88	**0.03**
*LA Strain*			
LA feature tracking reservoir strain (%)	-17.11 ± 3.52	-15.35 ± 4.24	**0.02**
LA feature tracking conduit strain (%)	-9.50 ± 2.63	-8.20 ± 3.02	**0.01**
LA feature tracking contractile strain (%)	-7.61 ± 2.16	-7.15 ± 2.65	0.30
LA peak positive strain rate	0.74 ± 0.24	0.66 ± 0.20	0.06
LA peak early negative strain rate	-0.92 ± 0.27	-0.74 ± 0.28	**0.001**
LA peak late negative strain rate	-0.87 ± 0.27	-0.81 ± 0.31	0.30
*LV parameters*			
LV ESV (ml)	71.87 ± 24.32	70.71 ± 21.03	0.78
LV EDV (ml)	171.28 ± 42.43	167.34 ± 37.90	0.59
LV stroke volume (ml)	99.40 ± 23.86	96.63 ± 27.78	0.55
LVEF (%)	58.49 ± 6.47	57.56 ± 8.76	0.50

Values are expressed as mean ± SD. AF, atrial fibrillation; AV, atrioventricular; CMR, cardiovascular magnetic resonance imaging; EDV, end diastolic volume; EF, ejection fraction; ESV, end systolic volume; LA, left atrial; LAEF, left atrial emptying fraction; LV, left ventricular; LVEF, left ventricular ejection fraction

### LA strain and strain rate in relation to LA sphericity

To gain insight into the association between LA sphericity and phasic strain, patients were dichotomized into groups according to the median LA sphericity (non-spherical LA ≤ 79.13% and spherical LA > 79.14%) (Table 2). Age and duration of AF were comparable between patients with a spherical and non-spherical LA. Patients with a more spherical LA had a significantly higher BMI (26.75 ± 3.78 kg/m^2^ vs. 24.82 ± 3.09 kg/m^2^, *P* = 0.002) (Table S1).

**Fig. 3:**
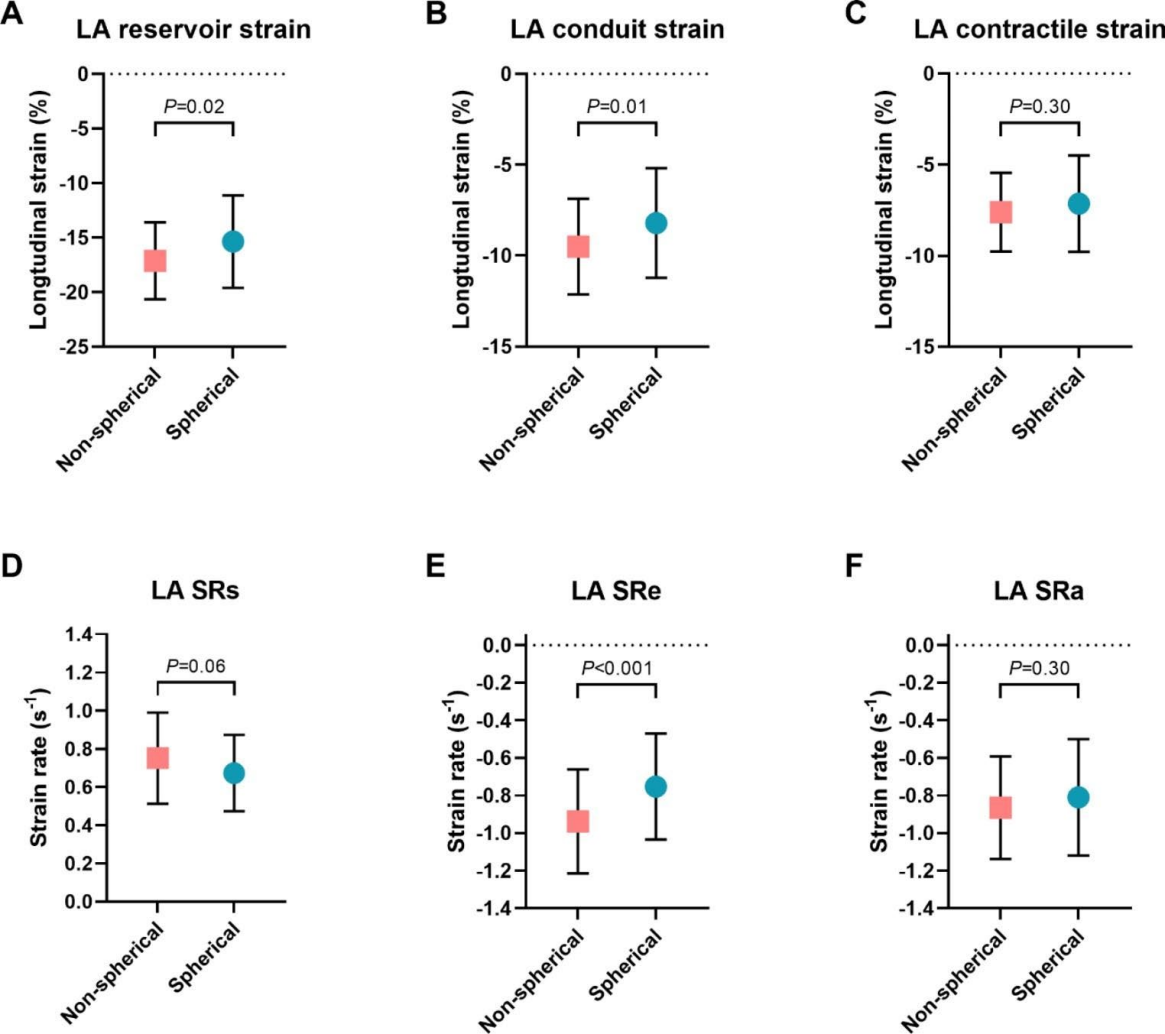
LA strain parameters in patients with a non-spherical and spherical LA geometry Difference in **A**) LA reservoir strain, **B**) LA conduit strain, and **C**) LA contractile strain between patients with a non-spherical and spherical LA geometry. Panel **D**, **E** and **F** demonstrate differences in LA strain rate between patients with a non-spherical and spherical LA geometry

**Fig. 4:**
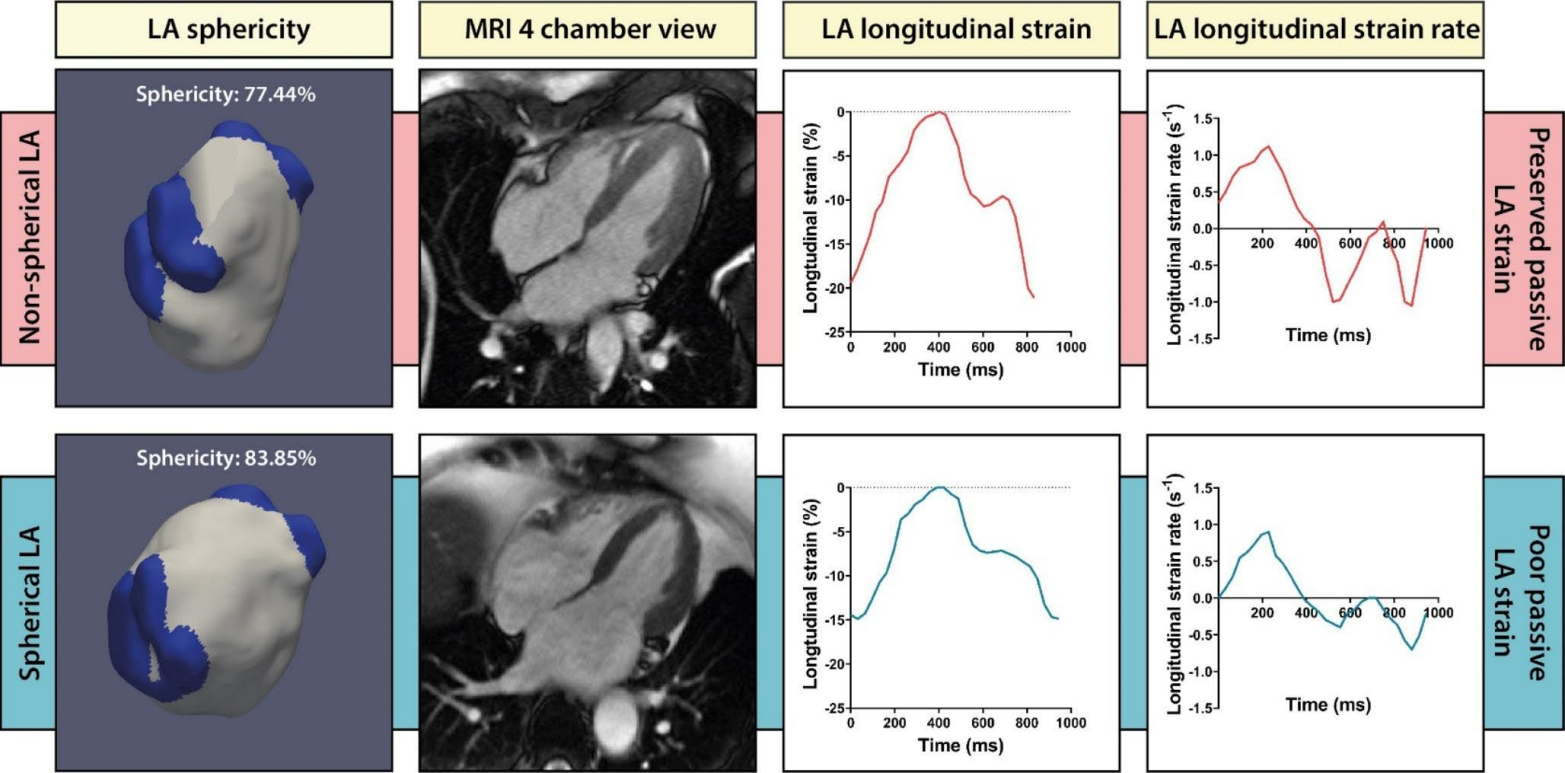
LA strain in an AF patient with a non-spherical and spherical LA geometry Illustrative example of a patient with a non-spherical LA **(A, B)** and spherical LA **(E,F)**, and the corresponding LA longitudinal strain curves and strain rate curves **(C,D,G,H)**.

Contractile strain was comparable between patients with a non-spherical and spherical LA (-7.61 ± 2.26% vs. -7.15 ± 2.65%, *P* = 0.30; Table 2; Fig. 3). Passive strain parameters, i.e. LA reservoir and LA conduit strain, were significantly impaired in patients with a more spherical LA (-15.35 ± 4.24% vs. -17.11 ± 3.52%, *P =* 0.02 and − 8.20 ± 3.01% vs. -9.50 ± 2.63%, *P* = 0.01, respectively). With regards to strain rate, LA early negative strain rate (SRe), representing conduit function with respect to time, was significantly reduced in patients with a spherical LA (-0.92 ± 0.27s^-1^ vs. -0.74 ± 0.28s^-1^, *P* = 0.001) (Fig. 4).

Multivariable linear regression analysis revealed that BMI, congestive heart failure, and 3D LAV were independently associated with LA reservoir strain (Table S2). A spherical LA was only independently associated with LA strain, when 3D LAV was left out of the model.

### LA pressure in relation to LA sphericity and LA strain

LA mean pressure was 10.12 ± 4.10mmHg. LA mean pressure had a significant but weak association with LA sphericity (r = 0.32, *P* < 0.01) (Figure S1), 3D LAV (r = 0.29, *P* = 0.01), and with LA strain (reservoir strain; r = 0.37, *P* = 0.001, conduit strain; r = 0.23, *P* = 0.05, contractile strain; r = 0.34, *P* < 0.01). LA strain rates were also correlated with LA pressure, LA positive strain rate; r=-0.24, *P* = 0.04, LA early negative strain rate; r = 0.28, *P* = 0.02, LA late negative strain rate; r = 0.31, *P* < 0.01. No significant association was found between LA v-wave pressure (16.07 ± 5.38mmHg) and LA sphericity, nor with LA strain indices.

**Fig. 5:**
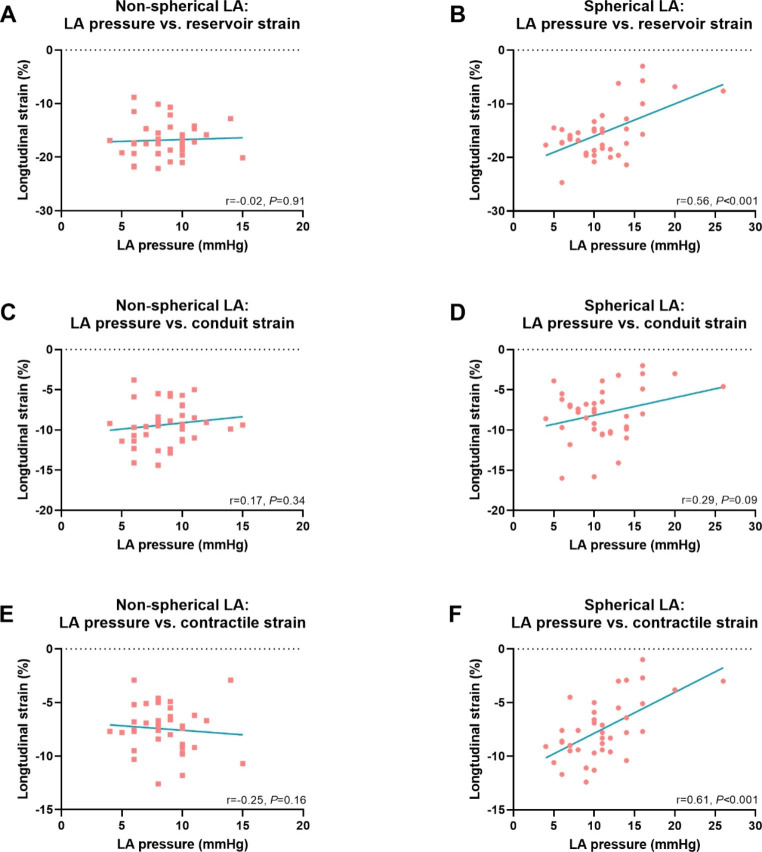
LA pressure – LA strain relation in patients with a non-spherical and spherical LA Correlation between LA pressure and LA reservoir strain in patients with a non-spherical LA **(A)** and patients with a spherical LA **(B)**. Correlation between LA pressure and LA conduit strain in patients with a non-spherical LA **(C)** and patients with a spherical LA **(D)**. Correlation between LA pressure and LA contractile strain in patients with a non-spherical LA **(E)** and patients with a spherical LA **(F)**. mmHg; millimeter of mercury

In patients with a spherical LA, LA mean pressure was correlated with LA reservoir strain and LA contractile strain (r = 0.56, *P* < 0.001 and r = 0.61, *P* < 0.001, respectively), while these correlations were absent in patients with a non-spherical LA (r=-0.02, *P* = 0.91 and r=-0.25, *P* = 0.16, respectively) (Fig. 5).

### Reproducibility

A total of 10 randomly selected patients underwent repeated review to assess intra- and inter-observer reliability (LH and PB). The ICC for inter-reader variability of LA sphericity measurements was 0.90 (95% confidence interval: 0.74–0.97). The ICC for intra-reader variability of LA sphericity measurements was 0.92 (95% confidence interval: 0.73–0.98).

## Discussion

This study investigated the impact of LA geometrical remodeling, expressed as sphericity, on LA functional parameters in patients with AF. The results indicate that passive LA function (defined as reservoir and conduit function) is impaired in patients with a spherical shaped LA, whereas contractile function was not different between patients with a spherical and non-spherical LA. LA volume however, was found to be an important determinant of LA strain, demonstrating to have a stronger association with LA strain than LA sphericity. A higher LA sphericity was associated with a higher LA pressure. In patients with a spherical LA, LA strain indices had a stronger correlation with mean LA pressure than in patients with a non-spherical LA. These observations suggest that LA geometrical and volumetric alterations, together with the intra-atrial pressure, impact LA phasic function.

### LA spherical remodeling in AF

Sphericity is a measure that expresses the comparison between an object and a sphere best fitted to that object [14]. In 2013, Bisbal et al. were the first to apply this concept to the LA, as a method to assess LA geometrical remodeling [6]. The demonstrated non-uniform dilatation during AF induced remodeling may result in an increasing LA sphericity. This can be explained from a mechanical perspective as a sphere is the best shape to resist hydrostatic pressure [15]. Patients with AF often have an increased intra-atrial pressure and volume, and consequently spherical remodeling would be a logical geometrical adaptation to cope with this pressure and volume (over)load. Subsequently, various studies marked LA sphericity as an important predictor of adverse ablation outcome and also found an independent association with prior thromboembolic events in AF patients [3, 6, 16]. On the other hand, recent studies could not reproduce these results and noticed that the degree of LA spherical dilatation may be restricted by thoracic dimensions and shape [15, 17, 18].

In the present study, a significant relationship was found between LA sphericity and volume, and LA sphericity and LA pressure. Furthermore, in line with previous studies, it was found that LA sphericity was higher in patients with hypertension as compared to patients without hypertension [18]. These findings support the hypothesis that a higher intra-atrial pressure may lead to increased spherical remodeling in order to accommodate the LA wall tension.

### LA sphericity in relation to strain

Patients with increased LA sphericity demonstrated an impaired passive LA function assessed using global longitudinal strain as compared to patients with a non-spherical LA. Besides LA passive strain, also early diastolic strain rate was markedly depressed in patients with a spherical LA. However, multivariable analysis demonstrated that LA sphericity was not independently associated with LA phasic strain, and LA volume was a stronger factor determining LA phasic strain.

Based on previous research, it can be postulated that spherical remodeling is associated with increased atrial stretch and that a spherical morphology may be linked to a more rigid and less compliant LA [2, 19, 20]. Potentially, excessive elevation of LA wall stress might lead to development of atrial fibrosis and consequently a reduced elastic recoil (i.e. atrial conduit function) [21]. This hypothesis is substantiated by Den Uijl and colleagues, demonstrating that a more sphere-shaped LA is associated with a higher degree of LA fibrosis [19]. In addition to previously published research, this study implies that both structural and geometrical remodeling may contribute to the decline of atrial function in AF patients, either mutually reinforcing or as self-contained processes [12].

In the present study, contractile strain was similar in patients with a spherical and non-spherical LA, suggesting that active LA contractile function is less affected by the geometry of the LA. Besides, as the passive LA function is impaired in patients with a spherical LA, LA active contractile function may serve as a compensatory mechanism to maintain proper LV filling and as a result is not different between patients with a spherical and non-spherical LA.

Interestingly, in patients with a spherical LA morphology, the relation between LA strain indices and LA pressure is stronger than in patients with a non-spherical LA. The most likely explanation may be that in patients with a non-spherical LA, the rather modest LA wall stress can compensate for an increase in LA pressure and therefore the LA function is preserved, even in the presence of (slightly) increased pressures. In patients with a spherical LA however, the persistent increased myocardial wall stress will result in already (over-)stretched myocytes, and therefore any further increase in pressure will ultimately lead to a deterioration in atrial function [22]. Hence, patients with a more spherical LA might be more prone to failure in atrial function.

Consequently, a spherical LA in combination with a preserved LA strain might be indicative for patients who might benefit from early AF ablation in order to achieve reverse atrial remodeling and prevent decline in atrial function [23]. In addition, a spherical LA in combination with a poor LA strain might indicate a more advanced stage of atrial remodeling identifying patients who might have a greater recurrence risk for AF after ablative treatment. Therefore, anatomical atrial characteristics such as shape combined with atrial phasic function can provide a more accurate patient specific clinical risk profile which may help in clinical decision making. This aims to enhance clinical results while decreasing expenses and preventing unnecessary procedures complications [24]. Nevertheless, this holistic model and patient specific approach needs to be evaluated in more detail in future studies.

### Limitations

The following limitations of the present study should be acknowledged. Firstly, our study used the most extensively validated method for sphericity calculations. However, segmentation and clipping of the PVs and LA appendage were manually performed. Potentially, these manual adjustments could lead to an inaccurate outcome. Nevertheless, intra-observer and inter-observer reliability analyses demonstrated excellent reproducibility for LA sphericity calculations. Secondly, intra-procedural LA pressure measurements were performed in only a subset of patients. These measurements were performed through the sheath after the PVI procedure was completed and before pulling back the trans-septal sheath. Ablation catheter cooling and consequent volume load during the PVI procedure might have influenced the invasively measured LA pressure. Moreover, this single measurement might not necessarily reflect patients’ chronic pressure loading condition. Also, the invasive pressure measurement could deviate a little from the actual LA pressure during CMR as there is an interval of approximately one month between the CMR exam and pressure measurements. Thirdly, another important factor in the understanding of LA wall stress is LA wall thickness. According to Laplace law, wall stress is inversely proportional to wall thickness [25]. Unfortunately, it was not possible to measure LA wall thickness by reason of the currently insufficient 3D CE-MRA resolution. Fourthly, a control group of healthy subjects is lacking and normal values for LA sphericity could not be assessed. Lastly, this study did not include post-ablation follow-up data and we could not assess whether LA sphericity is related to AF recurrence after ablation, or whether LA sphericity decreases in patients after successful AF ablation.

## Conclusions

LA remodeling is characterized by a confluence of changes in atrial geometry, volume and function. LA spherical morphology is associated with an impaired passive LA strain and strain rate, although this association was not independent from LA volume. In patients with a spherical LA, an increase in LA pressure is related with a deterioration in LA function while in patients with a normal, non-sphere shaped LA, LA function is largely preserved. Future studies should aim to clarify the clinical consequence of these findings.

### Electronic supplementary material

Below is the link to the electronic supplementary material.

Supplementary Material 1

## Data Availability

The datasets used and/or analyzed during the current study are available from the corresponding author upon reasonable request.

## Abbreviations

AF: atrial fibrillation
BMI: body mass index
CMR: cardiovascular magnetic resonance
EF: ejection fraction
LA: left atrial / left atrium
LA EF: left atrial emptying fraction
LAV: left atrial volume
LV: left ventricle
PVI: pulmonary vein isolation
RF: radiofrequency
