# Supplementary material for: Left atrial sphericity in relation to atrial strain and strain rate in atrial fibrillation patients
Source: Int J Cardiovasc Imaging. 2023 May 31;39(9):1753–63. doi: 10.1007/s10554-023-02866-2 (PMC10520187; doi:10.1007/s10554-023-02866-2)
Supplement: Supplementary file 1 — Supplementary Material 1 [file 10554_2023_2866_MOESM1_ESM.docx]

**SUPPLEMENTARY MATERIAL**

**Left Atrial Sphericity in Relation to Atrial Strain and Strain Rate in Atrial Fibrillation Patients**

Luuk H.G.A. Hopman, Pranav Bhagirath, Mark J. Mulder, Ahmet Demirkiran, Sulayman El Mathari, Anja M. van der Laan, Albert C. van Rossum, Michiel J. Kemme, Cornelis P. Allaart, Marco J.W. Götte

**Supplementary tables:**

**Table S1.** Differences in baseline characteristics in patients with a non-spherical LA and spherical LA

**Table S2.** Multivariable linear regression analysis of factors associated with left atrial reservoir strain

**Table S3.** Multivariable linear regression analysis of factors associated with left atrial conduit strain

**Table S4.** Multivariable linear regression analysis of factors associated with left atrial contractile strain

**Supplementary figures:**

**Figure S1.** LA sphericity - LA pressure relation in AF patients.

| **Table S1:** Differences in baseline characteristics in patients with a non-spherical LA and spherical LA | | | |
| --- | --- | --- | --- |
|  | **Non-spherical LA**  **(≤79.13%)**  ***n* = 64** | **Spherical LA (>79.14%)**  ***n* = 64** | ***P*-value** |
| Age, years | 58 ± 10 | 61 ± 10 | 0.06 |
| Male gender | 44 (69%) | 37 (58%) | 0.20 |
| BMI (kg/m^2^) | 24.82 ± 3.09 | 26.75 ± 3.78 | **0.002** |
| BSA | 2.03 ± 0.20 | 2.05 ± 0.22 | 0.68 |
| CHA_2_DS_2_-VASc score ≥2 | 18 (28%) | 28 (44%) | 0.08 |
| Hypertension | 19 (30%) | 25 (39%) | 0.26 |
| Diabetes mellitus | 1 (2%) | 4 (6%) | 0.17 |
| History of stroke/TIA | 1 (2%) | 3 (5%) | 0.31 |
| Congestive heart failure | 4 (6%) | 9 (14%) | 0.15 |
| Presence of mitral valve insufficiency | 20 (31%) | 22 (34%) | 0.75 |
| AF duration (months) | 39 ± 32 | 53 ± 53 | 0.36 |
| Values are expressed as number (percentage) or mean ± SD. AF, atrial fibrillation; BMI, body mass index; BSA, body surface area; CHA_2_DS_2_VASc, history of congestive heart failure, hypertension, diabetes mellitus, stroke/transient ischemic attack/prior thromboembolism, vascular disease, age and sex; LA, left atrial; TIA, transient ischemic attack. | | | |

| **Table S2:** Multivariable linear regression analysis of factors associated with left atrial reservoir strain | | | | | |
| --- | --- | --- | --- | --- | --- |
| **Variable** | **Coeff. B (unstandardized)** | **95% CI** | | **Beta (standardized)** | **P-value** |
|  |  | **Lower** | **Upper** |  |  |
| Age, years | -0.01 | -0.08 | 0.06 | -0.02 | 0.77 |
| Male gender | -0.54 | -1.93 | 0.85 | -0.06 | 0.44 |
| BMI (kg/m^2^) | -0.23 | -0.42 | -0.04 | -0.20 | **0.02** |
| Hypertension | 0.01 | -1.39 | 1.41 | 0.001 | 0.99 |
| Diabetes mellitus | 2.26 | -0.80 | 5.33 | 0.12 | 0.15 |
| History of stroke/TIA | 0.20 | -3.13 | 3.52 | 0.01 | 0.91 |
| Congestive heart failure | 3.77 | 1.47 | 6.06 | 0.26 | **<0.01** |
| Presence of mitral valve insufficiency | 0.80 | -0.55 | 2.15 | 0.09 | 0.24 |
| Spherical LA | 0.63 | -0.72 | 1.98 | 0.08 | 0.36 |
| 3D LA volume (ml) | 0.07 | 0.05 | 0.09 | 0.53 | **<0.001** |
| AF, atrial fibrillation; BMI, body mass index; LA, left atrial; TIA, transient ischemic attack. | | | | | |

| **Table S3:** Multivariable linear regression analysis of factors associated with left atrial conduit strain | | | | | |
| --- | --- | --- | --- | --- | --- |
| **Variable** | **Coeff. B (unstandardized)** | **95% CI** | | **Beta (standardized)** | **P-value** |
|  |  | **Lower** | **Upper** |  |  |
| Age, years | 0.06 | 0.01 | 0.12 | 0.20 | **0.03** |
| Male gender | 0.28 | -0.83 | 1.38 | 0.05 | 0.62 |
| BMI (kg/m^2^) | -0.08 | -0.23 | 0.07 | -0.10 | 0.28 |
| Hypertension | 0.08 | -1.03 | 1.20 | 0.01 | 0.88 |
| Diabetes mellitus | 1.74 | -0.69 | 4.17 | 0.13 | 0.16 |
| History of stroke/TIA | 1.01 | -1.62 | 3.65 | 0.07 | 0.45 |
| Congestive heart failure | 3.01 | 1.19 | 4.84 | 0.29 | **<0.01** |
| Presence of mitral valve insufficiency | 0.16 | -0.91 | 1.23 | 0.03 | 0.77 |
| Spherical LA | 0.50 | -0.57 | 1.57 | 0.09 | 0.36 |
| 3D LA volume (ml) | 0.03 | 0.01 | 0.05 | 0.28 | **<0.01** |
| AF, atrial fibrillation; BMI, body mass index; LA, left atrial; TIA, transient ischemic attack. | | | | | |

| **Table S4:** Multivariable linear regression analysis of factors associated with left atrial contractile strain | | | | | |
| --- | --- | --- | --- | --- | --- |
| **Variable** | **Coeff. B (unstandardized)** | **95% CI** | | **Beta (standardized)** | **P-value** |
|  |  | **Lower** | **Upper** |  |  |
| Age, years | -0.07 | -0.12 | -0.03 | -0.27 | **<0.01** |
| Male gender | -0.82 | -1.72 | 0.09 | -0.16 | 0.08 |
| BMI (kg/m^2^) | -0.15 | -0.27 | -0.02 | -0.21 | **0.02** |
| Hypertension | -0.08 | -0.99 | 0.84 | -0.02 | 0.87 |
| Diabetes mellitus | 0.52 | -1.47 | 2.51 | 0.04 | 0.60 |
| History of stroke/TIA | -0.82 | -2.98 | 1.35 | -0.06 | 0.46 |
| Congestive heart failure | 0.75 | -0.74 | 2.24 | 0.08 | 0.32 |
| Presence of mitral valve insufficiency | 0.64 | -0.24 | 1.52 | 0.12 | 0.15 |
| Spherical LA | 0.13 | -0.75 | 1.01 | 0.03 | 0.77 |
| 3D LA volume (ml) | 0.04 | 0.03 | 0.06 | 0.53 | **<0.001** |
| AF, atrial fibrillation; BMI, body mass index; LA, left atrial; TIA, transient ischemic attack. | | | | | |

**Supplemental Figure S1.** LA sphericity - LA pressure relation in AF patients


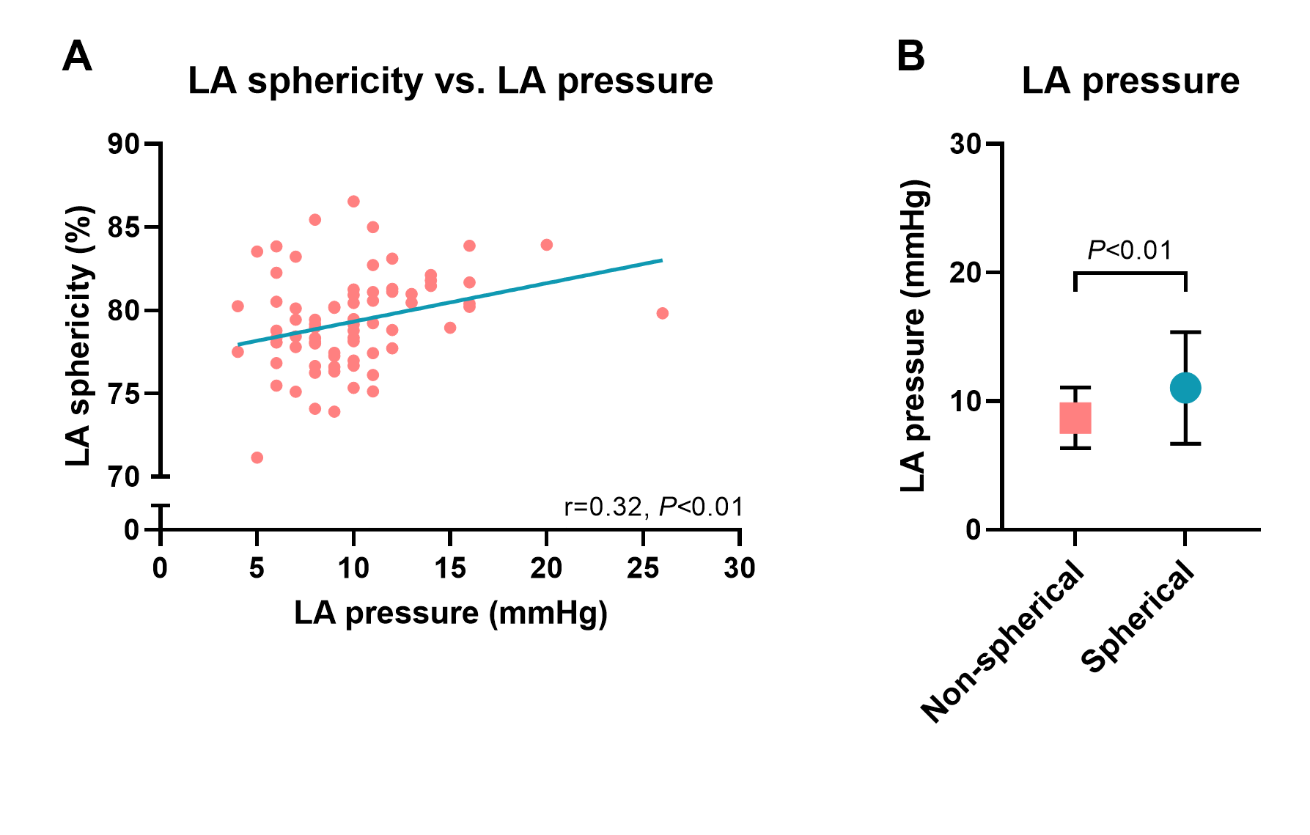


**(A)** Correlation between LA sphericity and LA pressure. **(B)** Difference in LA pressure between patients with a non-spherical LA and a spherical LA. mmHg; millimeter of mercury.
